# Epidemiological Investigation, Risk Factors, Spatial-Temporal Cluster, and Epidemic Trend Analysis of Pseudorabies Virus Seroprevalence in China (2017 to 2021)

**DOI:** 10.1128/spectrum.05297-22

**Published:** 2023-05-25

**Authors:** Pengfei Zhao, Yu Wang, Pengfei Zhang, Fen Du, Jianhai Li, Chaofei Wang, Rui Fang, Junlong Zhao

**Affiliations:** a State Key Laboratory of Agricultural Microbiology, Huazhong Agricultural University. Wuhan, Hubei, People’s Republic of China; b Hubei Center for Animal Disease Control and Prevention, Wuhan, Hubei, People’s Republic of China; c Wuhan Keweichuang Biotechnology Co., Ltd., Wuhan, Hubei, People’s Republic of China; National Chung Hsing University

**Keywords:** pseudorabies virus, seroprevalence, epidemiological investigation, risk factors, spatial-temporal clustering, epidemic trend analysis, China

## Abstract

Pseudorabies virus (PRV) is a double-stranded linear DNA virus capable of infecting various animals, including humans. We collected blood samples from 14 provinces in China between December 2017 and May 2021 to estimate PRV seroprevalence. The PRV gE antibody was detected using the enzyme-linked immunosorbent assay (ELISA). Logistic regression analysis identified potential risk factors associated with PRV gE serological status at the farm level. Spatial-temporal clusters of high PRV gE seroprevalence were explored using SaTScan 9.6 software. Time-series data of PRV gE seroprevalence were modeled using the autoregressive moving average (ARMA) method. A Monte Carlo sampling simulation based on the established model was performed to analyze epidemic trends of PRV gE seroprevalence using @RISK software (version 7.0). A total of 40,024 samples were collected from 545 pig farms across China. The PRV gE antibody positivity rates were 25.04% (95% confidence interval [CI], 24.61% to 25.46%) at the animal level and 55.96% (95% CI, 51.68% to 60.18%) at the pig farm level. Variables such as farm geographical division, farm topography, African swine fever (ASF) outbreak, and porcine reproductive and respiratory syndrome virus (PRRSV) control in pig farms were identified as risk factors for farm-level PRV infection. Five significant high-PRV gE seroprevalence clusters were detected in China for the first time, with a time range of 1 December 2017 to 31 July 2019. The monthly average change value of PRV gE seroprevalence was −0.826%. The probability of a monthly PRV gE seroprevalence decrease was 0.868, while an increase was 0.132.

**IMPORTANCE** PRV is a critical pathogen threatening the global swine industry. Our research fills knowledge gaps regarding PRV prevalence, infection risk factors, spatial-temporal clustering of high PRV gE seroprevalence, and the epidemic trend of PRV gE seroprevalence in China in recent years. These findings are valuable for the clinical prevention and control of PRV infection and suggest that PRV infection is likely to be successfully controlled in China.

## INTRODUCTION

Pseudorabies, also known as Aujeszky's disease, is caused by infection with the pseudorabies virus (PRV), a member of the *Varicellovirus* genus and the *Herpesviridae* family (1). Pigs are the natural host of PRV, but other species, including humans, mammals, carnivores, and rodents, can also be infected (2). PRV is a double-stranded linear DNA virus with a genome of approximately 143 kb, comprising at least 72 genes and measuring about 106 to 110 nm in diameter (3, 4). Clinical manifestations of PRV infection in pigs differ depending on age, as follows: high morbidity and mortality in 2-week-old suckling piglets, slow growth and dyspnea in fattening pigs, and reproductive disorders, mummification, and stillbirth in sows (5). The porcine reproductive and respiratory syndrome virus (PRRSV) is another significant infectious disease in pigs, causing severe immunosuppression and reproductive disorders in sows, similar to PRV (6, 7).

While PRV has been eradicated in North America and parts of Europe, it remains a major cause of reproductive disorders in sows in China (8). In the 1970s, the PRV Bartha-K61 vaccine strain was introduced into China and widely used for PRV prevention (1). However, in 2011, a PRV variant strain began spreading nationwide. Yang found this strain in 23 provinces, with 213 of 266 large-scale pig farms testing positive (9). On the basis of 108 articles published from 2011 to 2021, Tan et al. reported that 76,553 of the 256,326 blood samples tested positive for PRV gE antibody, representing a 29.87% positivity rate (10). In terms of molecular epidemiology, genotype 2 is the dominant strain in China, with a significantly different gene sequence from that of the commercial PRV vaccine (genotype 1) (11).

PRV gene-marked vaccines enable differentiation between vaccine immunity and natural infection by employing an enzyme-linked immunosorbent assay (ELISA) method based on the PRV gE gene (10), which allows for an epidemiological investigation of PRV infection by detecting PRV gE antibodies. African swine fever (ASF) is another severe porcine viral disease (12). The first ASF infection in China was reported in Shenyang City on 3 August 2018 (13). Following the ASF outbreak, most pig farms implemented stricter biosecurity measures, complicating blood collection from pig farms. Additionally, accurate estimates of the number of pigs raised in the study area are lacking. Therefore, this study employed a convenience sampling plan to collect pig blood samples from 545 pig farms across 14 provinces in China and to detect PRV gE antibodies assessing PRV seroprevalence in recent years. Logistic regression analysis was used to identify risk factors associated with PRV infection at the farm level. Spatial-temporal clustering of high PRV gE seroprevalence was assessed by combining the ELISA results of pig serum samples with the spatial coordinate data of pig farms. Finally, the established time series model simulated the epidemic trend of PRV gE seroprevalence. This research offers insights into the prevalence, risk factors, spatial-temporal clustering, and epidemic trend analysis of PRV. It provides valuable information for the clinical prevention and control of PRV infection at Chinese pig farms.

## RESULT

### Descriptive statistics of PRV gE seroprevalence.

**FIG 1:**
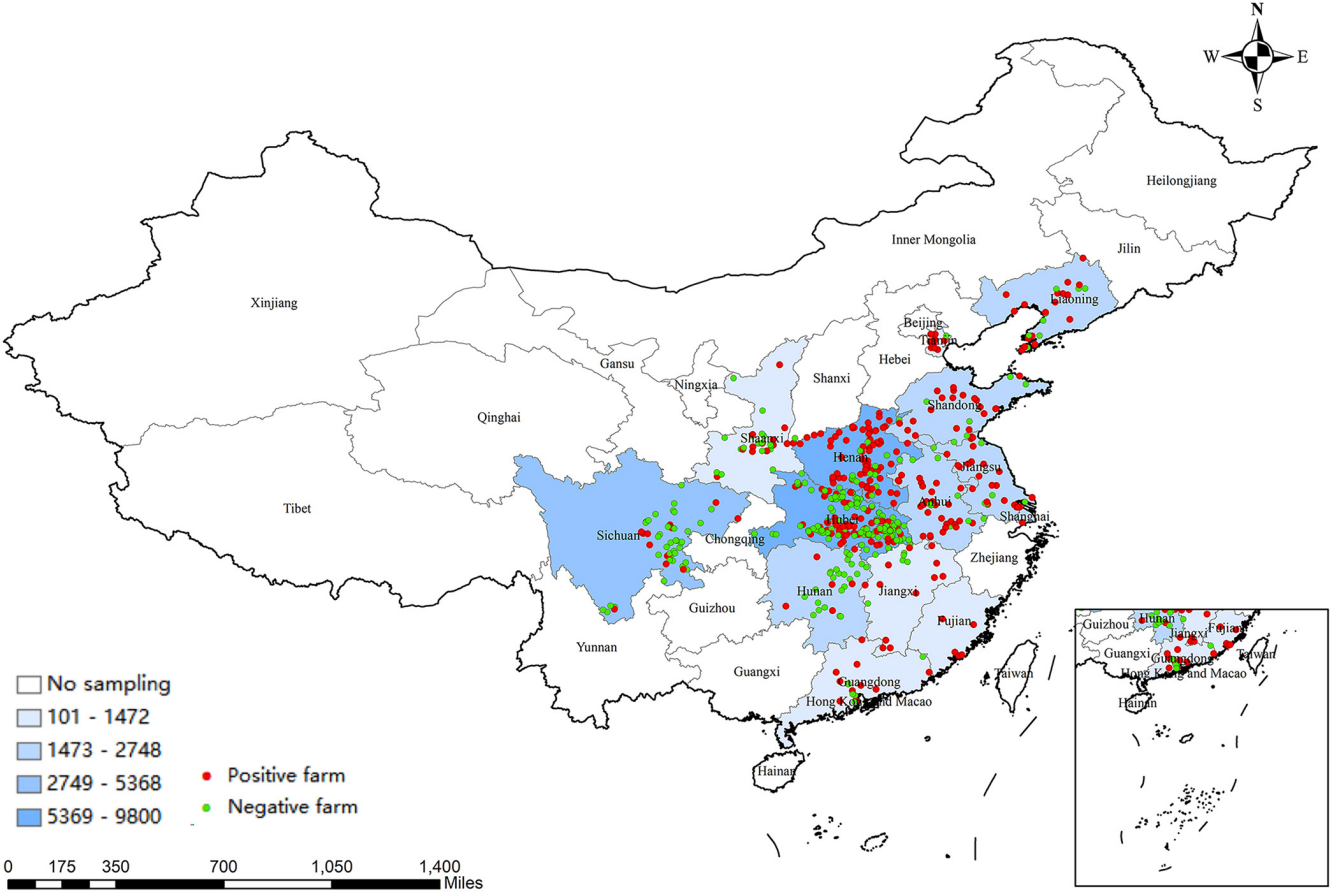
Distribution of samples and locations of pig farms collected from various provinces or cities of China from December 2017 to May 2021. The shade of square color represents the size of the sample quantity, red dots represent the pig farms of PRV gE antibody positive, and the green dots are PRV gE antibody negative.

**FIG 2:**
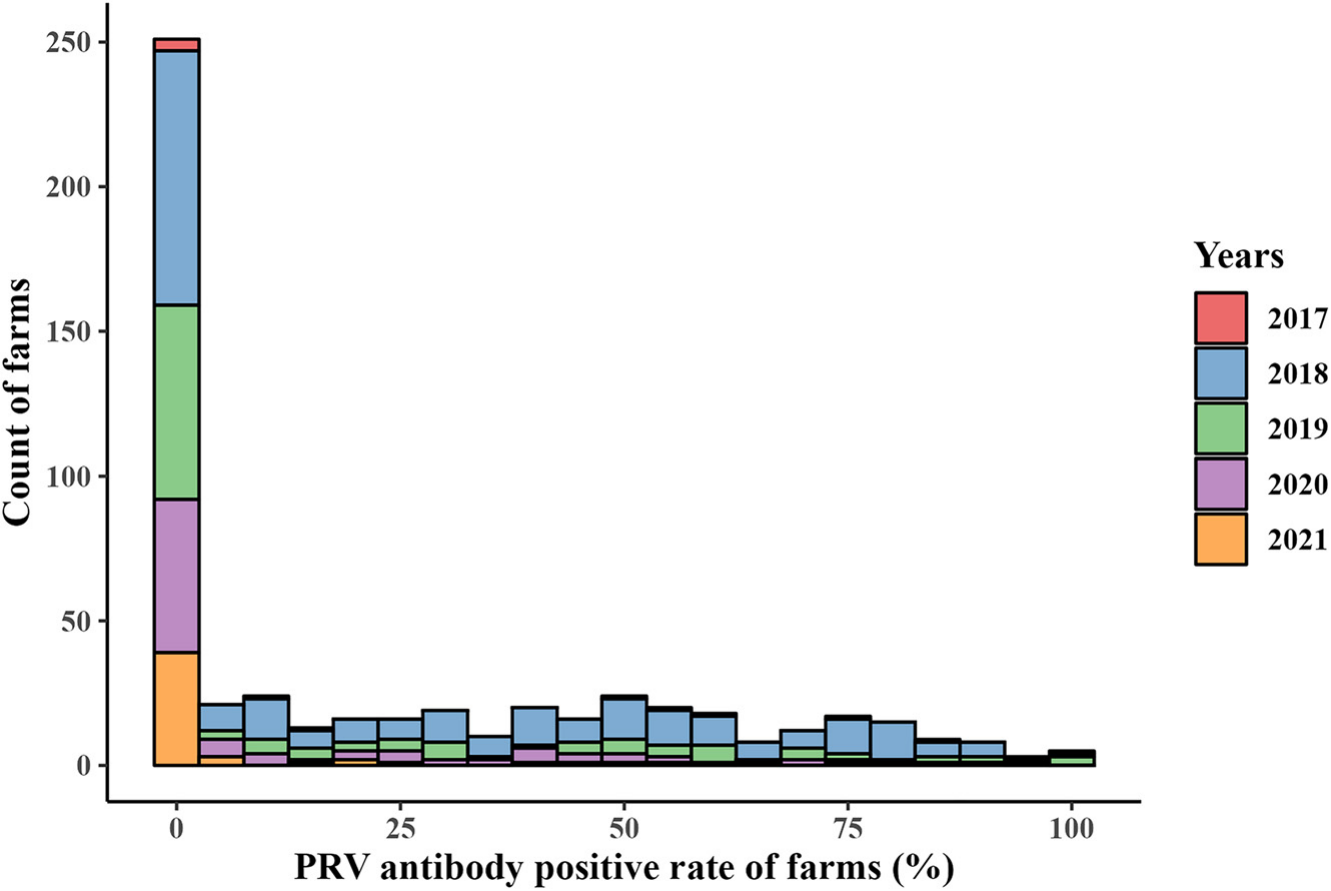
Histogram of PRV antibody-positive rates (%) in pig farms.

A total of 40,024 blood samples were collected from 545 pig farms in 14 provinces across China between December 2017 and May 2021 (Fig. 1). Of the 545 pig farms, 305 were positive for PRV gE antibody. The positivity rate of PRV gE antibody at the pig farm level was 55.96% (95% confidence interval [CI], 51.68% to 60.18%) (data not shown). The PRV gE seroprevalence at each pig farm ranged from 0% to 100% at the animal level (Fig. 2). The average positivity rate of PRV gE antibody across the 14 provinces in China was 25.04% (95% CI, 24.61% to 25.46%) (Table 1). The PRV gE antibody-positive rates in Fujian, Shandong Province, and Tianjin City were higher, at 61.94% (95%, CI, 57.84% to 65.91%), 53.98% (95% CI, 51.58% to 56.36%), and 73.49% (95% CI, 70.94% to 75.93%), respectively. In contrast, the positivity rates of PRV gE antibodies in Jiangxi, Shaanxi, Hunan Province, and Shanghai City were all lower than 10% (Table 1). The chi-square test value for PRV gE seroprevalence was 7,354.3 with a *P* value of <2.2 × 10^−16^, indicating significant variation among provinces (range, 5.43% to 73.49%) (Table 1). PRV gE antibody-positive rates were lower than 20% in growing and finishing pigs and boars. Similarly, the chi-square test value of PRV gE seroprevalence was 662.93 with a *P* value of <2.2 × 10^−16^, demonstrating significant differences among various pig categories (range, 16.02% to 30.74%) (Table 2).

**TABLE 1:** Prevalence of PRV gE antibody determined by Pearson chi-square test in provinces of China^*a*^

Province	No. of positive samples	Total no. of samples	Prevalence (%) (95% CI)
Anhui	997	2,748	36.28 (34.48–38.11)
Fujian	358	578	61.94 (57.84–65.91)
Guangdong	161	1,138	14.15 (12.17–16.31)
Henan	3,194	7,942	40.22 (39.14–41.30)
Hubei	1,249	9,800	12.74 (12.09–13.42)
Hunan	212	2,138	9.92 (8.68–11.26)
Jiangsu	649	2,042	31.78 (29.77–33.85)
Jiangxi	80	1,472	5.43 (4.33–6.72)
Liaoning	745	1,858	40.10 (37.86–42.37)
Shandong	923	1,710	53.98 (51.58–56.36)
Shanghai	30	318	9.43 (4.46–13.19)
Shaanxi	115	1,671	6.88 (5.72–8.20)
Sichuan	396	5,368	7.38 (6.69–8.11)
Tianjin	912	1,241	73.49 (70.94–75.93)
Total	10,021	40,024	25.04 (24.61–25.46)

a The chi-square test value for PRV gE seroprevalence in different provinces was 7,354.3 with a *P* value of <2.2 × 10^−16^. *, *P* < 0.05; **, *P* < 0.01; ***, *P* < 0.001.

**TABLE 2:** Prevalence of PRV gE antibody determined by Pearson chi-square test in different stages of pigs^*a*^

Pig stage	No. of positive samples	Total no. of samples	Prevalence (%) (95% CI)
Piglets (≤21 days)	1,221	3,972	30.74 (29.31–32.20)
Weaned piglets (22 to 70 days)	2,185	7,203	30.33 (29.27–31.41)
Growing-finishing pigs (≥71 days)	979	6,104	16.02 (15.11–16.97)
Replacement gilts	846	4,110	20.58 (19.36–21.85)
Multiparous sows (≥1 parity)	3,937	14,000	28.12 (27.38–28.87)
Boars	853	4,635	18.40 (17.30–19.55)

a The chi-square test value for PRV gE seroprevalence in different pig stages was 662.93 with a *P* value of <2.2 × 10^−16^.

### Risk factor analysis associated with PRV serological status at the pig farm level.

In the univariate logistic regression model (Table 3), variables such as pig farm size, geographical location of pig farm, topography of pig farm, ASF outbreak, and purification and immunity status of PRRSV in pig farms had *P* values of less than 0.1. Consequently, these variables were selected for the multivariate logistic regression model. The multivariate logistic analysis (Table 4) identified four risk factors associated with PRV serological status in pig farms, excluding the pig farm size variable. Pig farms in Central China, Northwest China, and Southwest China were less likely to be infected with PRV than those in Eastern China. The likelihood of PRV infection in pig farms in plain areas was 3.782 (95% CI, 2.327 to 6.333) times higher than in mountainous or hilly areas. The odds ratio (OR) value of pig farms before the ASF outbreak was 1.667 (95% CI, 1.075 to 2.606) times higher than for pig farms after the outbreak. The likelihood of PRV infection in PRRSV-positive farms with PRRSV vaccine immunity was 31.540 (95% CI, 7.813 to 240.094) times higher than in PRRSV-negative farms. Additionally, PRV infection in PRRSV-positive farms without PRRSV vaccine immunity also showed a higher probability than in PRRSV-negative farms (OR, 26.238; 95% CI, 6.499 to 199.413).

**TABLE 3:** Univariate logistic analysis of risk factors associated with PRV serological status of pig farm levels

Variable and category	OR (95% CI)	*P* value
Season		
Autumn	1 (reference)	
Spring	0.741 (0.477–1.148)	0.18
Summer	1.095 (0.658–1.830)	0.728
Winter	0.990 (0.606–1.620)	0.968
Farm size		
Small (<100 sows)	1 (reference)	
Medium (100 to 500 sows)	0.631 (0.413–0.960)	0.032
Large (>500 sows)	0.705 (0.452–1.093)	0.12
Geographic location of pig farms		
Eastern China	1 (reference)	
Central China	0.390 (0.235–0.631)	1.73 × 10^−4^
North China	5.127 (0.967–94.862)	0.122
Northeast China	0.732 (0.277–2.090)	0.541
Northwest China	0.161 (0.060–0.403)	1.56 × 10^−4^
South China	0.627 (0.216–1.967)	0.399
Southwest China	0.128 (0.060–0.262)	4.65 × 10^−8^
Topography of pig farms		
Hills or mountains	1 (reference)	
Plains	3.309 (2.237–4.966)	3.79 × 10^−9^
Outbreak of ASF		
Yes	1 (reference)	
No	1.721 (1.172–2.547)	6.03 × 10^−3^
Purification and immunity status of PRRSV in pig farms		
PRRSV-negative farm	1 (reference)	
PRRSV-positive farm with no PRRSV vaccines immunity	17.122 (4.989–107.553)	1.33 × 10^−4^
PRRSV-positive farm with PRRSV vaccine immunity	20.143 (5.830–126.928)	5.69 × 10^−5^

**TABLE 4:** Multivariable logistic analysis of risk factors associated with PRV serological status of pig farm levels

Variable and category	OR (95% CI)	*P* value
Geographic location of pig farm		
Eastern China	1 (reference)	
Central China	0.457 (0.266–0.768)	3.69 × 10^−3^
North China	4.852 (0.693–111.535)	0.191
Northeast China	0.918 (0.314–2.962)	0.879
Northwest China	0.065 (0.022–0.178)	2.32 × 10^−7^
South China	1.17 (0.360–4.342)	0.801
Southwest China	0.158 (0.070–0.340)	4.09 × 10^−6^
Topography of pig farm		
Hills or mountains	1 (reference)	
Plains	3.782 (2.327–6.333)	1.74 × 10^−7^
Outbreak of ASF		
Yes	1 (reference)	
No	1.667 (1.075–2.606)	2.34 × 10^−2^
Purification and immunity status of PRRSV in pig farm		
PRRSV-negative farm	1 (reference)	
PRRSV-positive farm with no PRRSV vaccines immunity	26.238 (6.499–199.413)	9.01 × 10^−5^
PRRSV-positive farm with PRRSV vaccine immunity	31.54 (7.813–240.094)	3.58 × 10^−5^

### Spatial-temporal clustering of high PRV gE seroprevalence.

**FIG 3:**
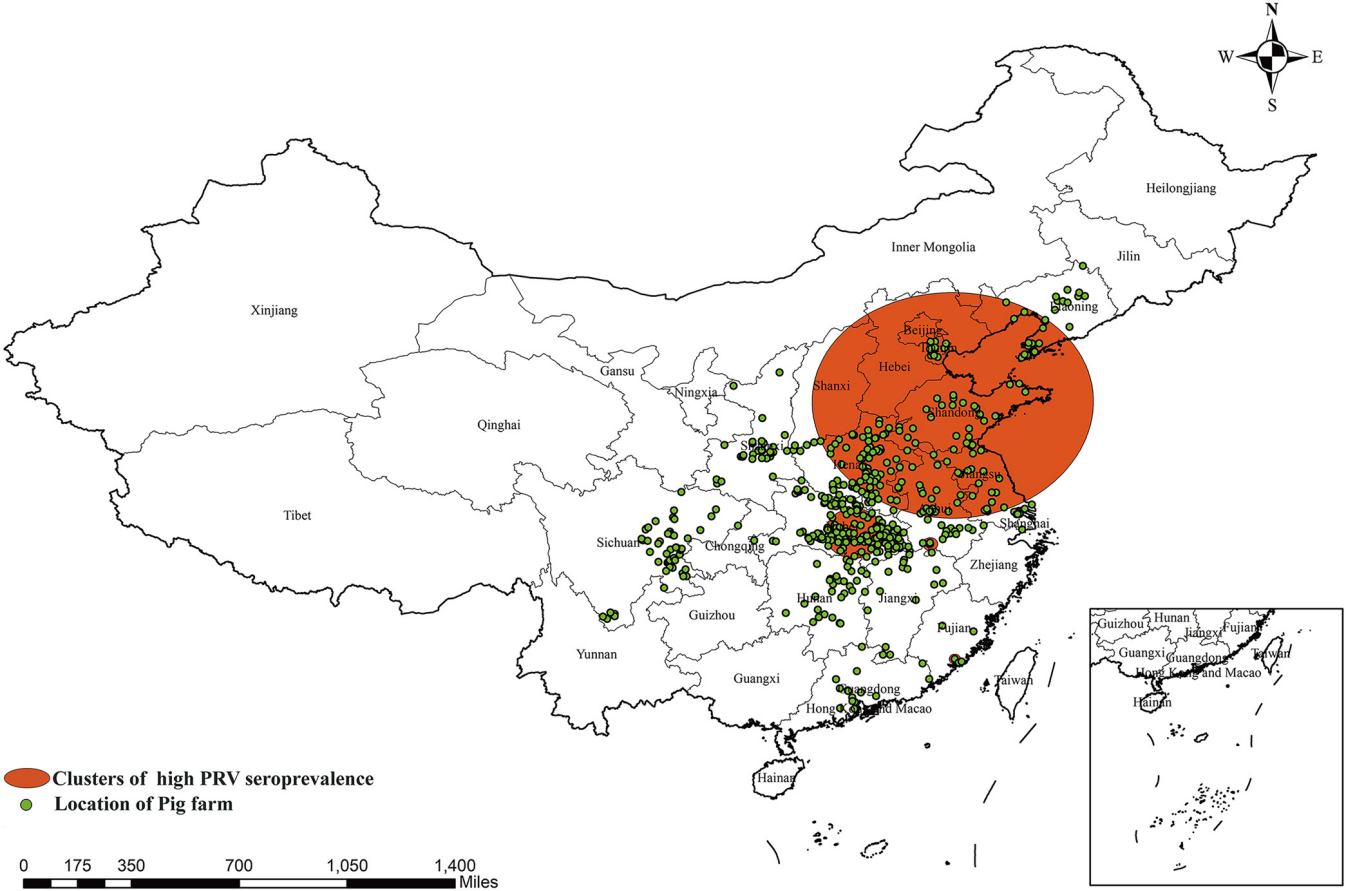
Significant spatial clusters (*P* < 0.05) of high PRV gE seroprevalence in China from December 2017 to May 2021 with a maximum window size of 25% of the population at risk. Clusters 2 and 5 can not be clearly displayed on the map due to the small area.

Figure 3 and Table 5 display the five clusters of high PRV gE seroprevalence identified in China between December 2017 and May 2021. The largest cluster is at 36.657313 N, 118.008476 E, with a 588.4-km radius, spanning the time range from 1 December 2017 to 31 July 2019 and exhibiting a relative risk value of 3.13. The second cluster, located at 24.745608 N, 118.106325 E, has a radius of 26.15 km and occurred between 1 December 2017 and 30 June 2018, with a relative risk value of 3.41. The third cluster, the second-largest area, has a radius of 130.14 km and is situated at 30.646757 N, 113.374779 E. This cluster's time range is from 1 May 2018 to 31 July 2018, with a relative risk value of 2.78. The fourth cluster is positioned at 30.148515 N, 116.952775 E, with a radius of 33.43 km. The smallest cluster is located at 30.619455 N, 104.762949 E, with a radius of 11.58 km. The time ranges of the last two clusters are from 1 July 2018 to 30 September 2019 and 1 February 2018 to 31 August 2018, respectively. Their corresponding relative risk values are 3.35 and 2.58.

**TABLE 5:** Spatial-temporal clusters of PRV gE seroprevalence in China from 2017 to 2021

Cluster	Coordinates	Cluster radius (km)	Time range (yr/mo/day)	Relative risk	*P* value
1	36.657313 N, 118.008476 E	588.43	2017/12/1–2019/7/31	3.13	<10^−17^
2	24.745608 N, 118.106325 E	26.15	2017/12/1–2018/6/30	3.41	<10^−17^
3	30.646757 N, 113.374779 E	130.14	2018/5/1–2018/7/31	2.78	<10^−17^
4	30.148515 N, 116.952775 E	33.43	2018/7/1–2019/9/30	3.35	<10^−17^
5	30.619455 N, 104.762949 E	11.58	2018/2/1–2018/8/31	2.58	<10^−17^

### Time series model building and epidemic trend analysis of the PRV gE seroprevalence.

**FIG 4:**
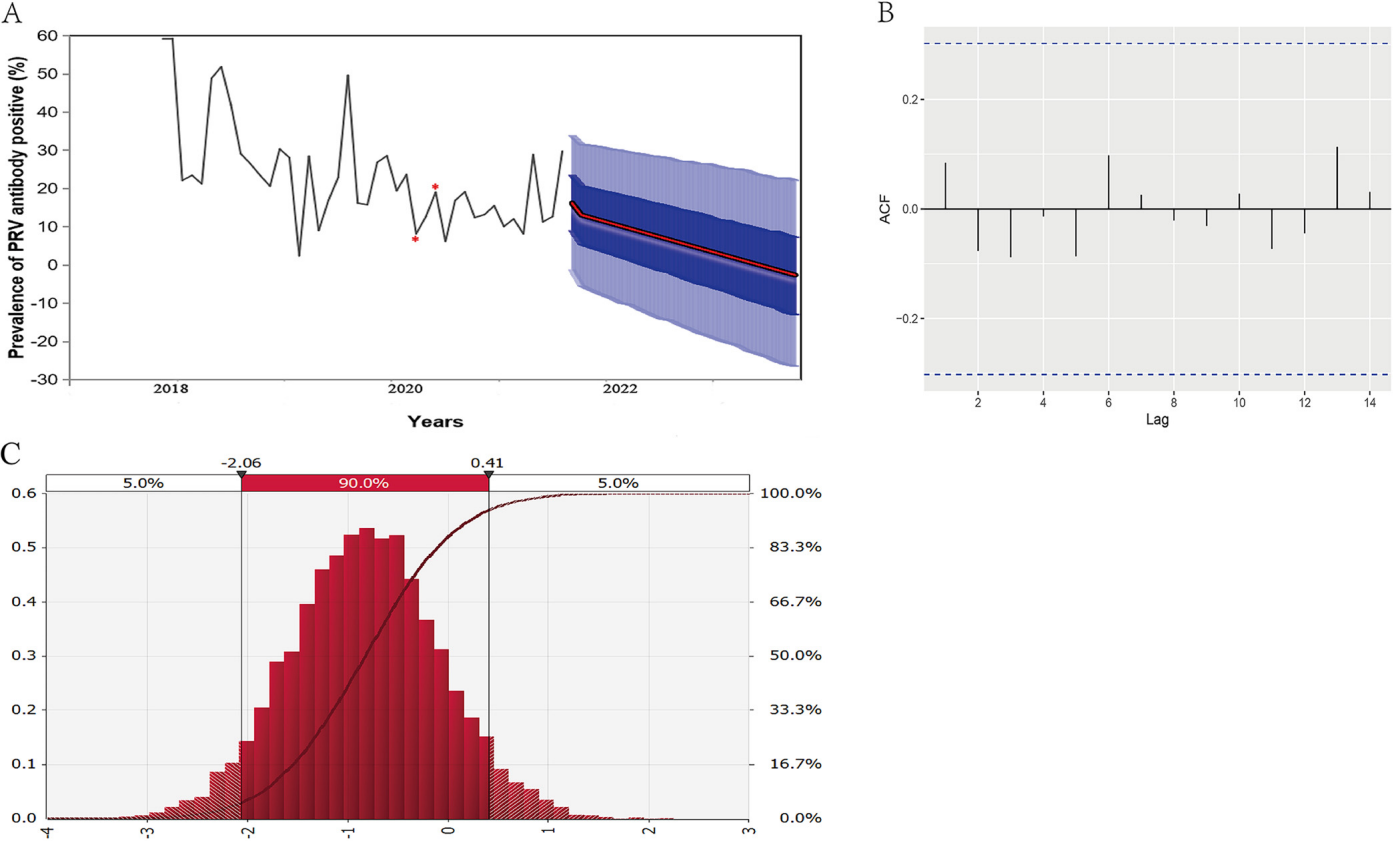
PRV MA2 model of PRV antibody-positive rate. (A) Positive rate and prediction values (%) of PRV antibody-positive rate. The red line is the average prediction value of the PRV antibody-positive rate (%). The dark blue area indicates the 50% CI, and the light blue area represents the 95% CI. Red stars denote interpolated values of missing PRV prevalence due to COVID-2019 in February and March 2020. (B) Autocorrelation plot of PRV antibody-positive rate. The dashed lines are 5% significance limits for the autocorrelation. (C) Simulated sampling distribution diagram of the monthly average change rate of PRV seroprevalence. The red line is probability density cumulative curve. The red histogram is the frequency distribution of varied monthly average change rates.

The PRV moving average 2 (MA2) model has been successfully established with the lowest Akaike information criterion (AIC) value of 320.7423 (data not shown) (Fig. 4A), exhibiting a clear downward trend in PRV gE seroprevalence. The autocorrelation residuals are also within the detection lines, indicating that the constructed model is reasonable and stable (Fig. 4B). Consequently, simulated sampling was performed to estimate the monthly average change rate of PRV gE seroprevalence on the PRV MA2 model. Figure 4C displays the sampling distribution diagram of the monthly average change rate in PRV gE seroprevalence, with a 90% sampling interval of −2.06% to 0.41%. The mean value of the monthly average change rate is −0.826% with a standard deviation (SD) of 0.747. Additionally, it is calculated that the probability of the monthly average change rate of PRV gE seroprevalence being negative is 0.868, while the probability of it being positive is 0.132 (Table 6).

**TABLE 6:** Simulated sampling statistics of the monthly average change rate of PRV gE seroprevalence for model MA2^*a*^

Statistic	Value
Monthly avg change rate (%) (mean ± SD)	−0.826 ± 0.747
Probability of positive change	0.132
Probability of negative change	0.868

a MA, moving average.

## DISCUSSION

To assess the current epidemic situation of PRV gE seroprevalence in China, we performed a cross-sectional survey by collecting pig blood samples for PRV gE antibody detection. Our study involved 40,024 blood samples from 545 large, medium, and small pig farms across 14 provinces in China. As PRV gene-marked attenuated vaccines (artificially or naturally lacking the PRV gE gene) are widely used for PRV infection prevention and control, detection of the PRV gE antibody can indicate a natural infection in pig farms (14). We identified 305 pig farms with PRV gE antibody-positive results, revealing a 55.96% positivity rate (95% CI, 51.68% to 60.18%) at the pig farm level. This outcome aligns with the study performed by Xia et al., which reported a 67.6% PRV gE antibody-positive rate (95% CI, 57.0% to 77.0%) at the pig farm level (15). Furthermore, the overall PRV gE seroprevalence at the animal level was 25.04% (95% CI, 24.61% to 25.46%). Because accurate data on pig feeding numbers, pig farm numbers, and PRV gE prevalence in China are unavailable, our study utilized a convenience sampling method to select pig farms and collected serum samples according to the sampling procedure described below. However, after the ASF outbreak, most pig farms began implementing closed management, which resulted in collection of samples only by farmers themselves rather than on-site sampling. However, some farmers might be more inclined to collect samples from sick or weak pigs to determine whether PRV infection in their pig farms, potentially resulting in a slightly elevated PRV gE seroprevalence. Additionally, the herd structure of the farms selected by the convenience sampling method was inconsistent. Not all farms contained all pig categories (piglets, weaned pigs, growing and finishing pigs, replacement gilts, and boars), which might also affect the PRV gE seroprevalence in pig farms since there are significant differences in PRV gE seroprevalence among various pig categories (Table 2).

Multivariate logistic regression analysis revealed that the possibility of PRV infection in pig farms in plain areas was 3.782 (95% CI, 2.327 to 6.333) times higher than in mountainous or hilly areas. Pig farms in mountainous or hilly regions benefit from the natural biosafety barrier created by the terrain. Ruiz-Fons et al. found no significant statistical correlation between the PRV seroprevalence in wild boars and domestic pigs, suggesting that wild boars or other wild animals do not impact PRV transmission (16). Pig farms sampled after the ASF outbreak demonstrated a lower likelihood of PRV infection than those before the outbreak, possibly due to improved biosafety management. The pig farm size was not identified as a risk factor associated with PRV serological status in pig herds, consistent with Bouma et al.’s conclusion that pig herd size does not influence PRV transmission speed (17). Pig farms in Central China, Northwest China, and Southwest China have a lower likelihood of PRV infection than those in Eastern China, which is in line with Liu et al.'s research on PRV prevalence in Chinese fattening pig farms from 2013 to 2016, which reported and found significant regional variations in PRV gE antibody-positive rates (18). PRRSV-positive farms are approximately 20 to 30 times more likely to be infected with PRV than PRRSV-negative farms, potentially due to PRRSV's immunosuppressive effects, which can significantly reduce pig immunity and increase susceptibility to infection (6). Coinfections of PRV and other pathogens are common in domestic pig farms. Ma et al. investigated PRV prevalence in Shandong Province from 2015 to 2018 and found the highest coinfection rate of PRV and porcine circovirus 2 (PCV2) to be 35.0% (19). Based on the risk factor analysis, we recommend establishing pig farms in mountainous or hilly areas and strengthening PRRSV purification efforts for PRV prevention and control. However, for PRRSV-positive farms, comprehensive biosafety prevention and control measures should be prioritized over PRRSV immunization.

Allepuz et al. conducted a spatial analysis of PRV incidences in pig farms in Catalonia, Spain, between 2003 and 2007, identifying PRV infection aggregations in sow and fattening pig farms (20). Berke et al. detected two spatial aggregations of PRV infection in Germany, with radii of 2.6 km and 1.7 km and relative risk values of 2.4 and 3.3 (21). However, research on spatial-temporal clusters of PRV in China is scarce. Therefore, we analyzed the spatial-temporal cluster of high PRV gE seroprevalence in China, identifying five significant clusters for the first time between 1 December 2017 and 31 July 2019. These high PRV gE seroprevalence clusters were geographically close to the areas with high PRRSV seroprevalence identified by Zhao et al. (22), further suggesting a possible link between PRV and PRRSV infection. Nonetheless, the regional area and time range of cluster 1 (Table 5) are extensive, indicating a potentially more precise cluster distribution of disease occurrence in this area and period. Future research should increase the number of sampled pig farms in this area to narrow down and accurately pinpoint the high PRV gE seroprevalence aggregation area.

A clear downward trend of PRV gE seroprevalence is evident from the established PRV MA2 model. Similarly, Liu et al. found a decreased positive rate of PRV gE antibody from 2013 to 2016 (22.17% to 13.14%) (18). Chen et al. analyzed 35,796 serum samples collected in Henan province from 2019 to 2021 and observed that the positivity rate of PRV gE antibody dropped from 25% to 16.69% (23). These epidemiological findings are consistent with our model results. From December 2017 to December 2019, prior to the COVID-19 outbreak, an average of 1,179 blood samples were collected per month. Following the COVID-19 outbreak, from January 2020 to May 2021 (excluding February and March 2020), the monthly average dropped to 703 samples (data not shown). Before the ASF outbreak (from December 2017 to August 2018), the monthly average was 1,432 blood samples; after the outbreak, this number decreased to 875 (data not shown). The ASF outbreak had a more significant impact on sample collection than the COVID-19 pandemic. Nevertheless, the PRV gE seroprevalence consistently exhibited a downward trend during both periods (Fig. 4A). Thus, although our sampling quantity declined due to various factors, the monthly sample collection remained relatively large and representative, accurately reflecting the evolving trend of PRV gE seroprevalence. Using Monte Carlo simulations, we quantitatively analyzed the epidemic trend of PRV gE seroprevalence. The mean value of the monthly average change rate of PRV gE seroprevalence was −0.826%, indicating an average monthly decline of 0.826%. Additionally, the probability of a decrease in monthly PRV gE seroprevalence was 0.868 (monthly average change rate, <0). These findings suggest that PRV may eventually be eradicated from China. Moreover, China's current PRV prevention and control strategy is reasonable and practical, primarily involving PRV vaccine immunization and detection and elimination of gE antibody-positive pigs (5).

### Conclusions.

We conducted an epidemiological investigation of PRV gE seroprevalence in China from 2017 to 2021, collecting 40,024 blood samples from 545 pig farms across 14 provinces to detect PRV gE antibodies by using ELISA. The overall PRV gE seroprevalences were 25.04% (95% CI, 24.61% to 25.46%) at the animal level and 55.96% (95% CI, 51.68% to 60.18%) at the pig farm level. Moreover, significant differences in PRV gE seroprevalence existed among provinces and pig categories. Through multivariate logistic regression analysis, we identified the risk factors associated with PRV serological status in pig farms as geographical location of the pig farms, topography of pig farms, outbreak of ASF, and purification and immunity status of PRRSV in pig farms. For the first time, we detected five significant clusters of high PRV gE seroprevalence in China, with a time range from 1 December 2017 to 31 July 2019. The epidemic trend of PRV gE seroprevalence is a downward trend with a monthly average change rate of 0.826%. The probability of a decrease in the monthly PRV gE seroprevalence is 0.868, while the probability of an increase is 0.132. Our research findings fill the knowledge gaps regarding the prevalence, risk factors, spatial-temporal clusters, and epidemic trends of PRV gE seroprevalence in China, providing valuable insights for the clinical prevention and control of PRV infection.

## MATERIALS AND METHODS

### Study area and population.

The study area encompassed seven major geographical divisions in China, namely, Central China (Henan, Hubei, and Hunan Province), East China region (Shandong, Jiangsu, Anhui, Jiangxi, Fujian Province, and Shanghai City), Northeast China region (Liaoning Province), South China (Guangdong Province), Southwest China (Sichuan Province), Northwest China (Shaanxi Province), and North China (Tianjin City). Spanning a longitude range of 97°20′ to 126°00′E and a latitude range of 18°10′ to 43°30′N, the study area covers approximately 2.3 million km^2^. This region features diverse monsoon climates with annual average temperatures ranging from 3°C to 28°C and various topographical landscapes, including plateaus, mountains, plains, hills, and basins. Geographical coordinates of pig farms were obtained using the Baidu Map (https://map.baidu.com/). Blood samples were collected from various pig herds, including piglets (from birth to 21 days), weaned pigs (22 to 70 days), growing and finishing pigs (over 70 days), replacement gilts (≥1 parity), and boars (22, 24).

### Sampling design.

A commercial PRV/ADV gE antibody detection kit (IDEXX, Switzerland) was utilized to detect the PRV gE antibody, with a sensitivity of 96.7% and specificity of 99.8%, in accordance with the manufacturer’s instructions (15). We followed a two-stage sampling strategy in this study. In the first stage, an assumed herd-level prevalence of 50%, with a 95% CI, desired precision of 5%, and large population (unknown), was used to calculate the herd-level sample size using Epitools (https://epitools.ausvet.com.au/) (25), which resulted in a minimum sample size of 385 farms. The second sampling stage assumed an expected minimum prevalence of 10%, a 95% CI, and a median herd size of 500 to calculate the number of animals sampled per farm, using the following formula (26):

$$n=\left(1-{\left(\text{α}\right)}^{1/D}\right)\left(N-\frac{D-1}{2}\right)$$

where *n* is the required sample size, α is a value of 1 minus the confidence level of disease prevalence (usually 0.05), *D* is the estimated minimum number of diseased animals in the pig farms (population size × minimum expected prevalence), and *N* is the population size.

This required a minimum sample size of 28 animals per farm. If the total number of feeding pigs on the farm is less than 28, serum is collected from all animals. Besides, there are different pig categories in the pig farms. Therefore, based on a 50% prevalence of PRV gE antibody in the animal population, a 95% CI, and a desired precision of 5%, we determined that a minimum of 408 animals must be sampled in each pig category. Owing to closed management of pig farms caused by the ASF outbreak in China and budget constraints, we employed a sampling method to select the sampled pig farms and collected samples based on pig farm information acquired through a third-party testing platform (27). At the beginning of the month, we contacted the farmers for advice on sample collection based on the farm information obtained the previous month. After obtaining the consent of the farmers, the blood samples were collected on-site (in Hubei province) or by resident veterinarians (outside Hubei province) and then sent via cold-chain transportation. The farms already selected for serum sampling would not be included again. The sampling ratio of each pig category within each farm was determined according to the percentage of each pig category at the farm. Concurrently, background information on the sampled pig farm and collected pig blood samples was documented through phone or face-to-face interviews with farmers. Critical information included sampling time, sample number, farm location, pig farm size, farm topography, pre- or post-ASF outbreak status, and PRRSV infection and immunity. For the ASF outbreak variable, because our experimental period covered just the entire stage of ASF outbreak in China, one of purposes of this study was to compare domestic PRV gE seroprevalence before and after the ASF outbreak. These collected serum samples were also used concurrently in another PRRSV prevalence survey. Meanwhile, the coinfections of PRRSV with other viruses were common clinically. Therefore, we also added the PRRSV infection and immunity variable to the study design. The ASF outbreak and farm topography variables were treated as binary variables. Farm size was categorized into three groups based on the number of sows: small (≤100), medium (100 to 500), and large (≥500) (28).

### Sample collection.

The selected pigs were restrained, and 5 to 10 mL of blood was collected from the anterior vena cava using a sterile needle and vacutainer. The collected blood was then sent to the laboratory via cold-chain transportation. All processes involved in animal handling complied with relevant regulations and animal welfare requirements in China, as well as the relevant standards of the Huazhong Agricultural University Ethics Committee (HZAUSW-2022-0008). Subsequently, the blood was centrifuged at 3,000 rpm for 5 min. The supernatant serum was transferred to a sterile centrifuge tube and stored at −20°C for later use.

### PRV gE antibody detection.

The experimental operation was carried out according to the kit instructions. After 2-fold dilution of the tested sample, negative, and positive controls with sample diluent, 100 μL of solution was added to the antigen-coated plate and incubated at 18 to 26°C for 60 min. The wells were washed with 300 μL of wash solution, with the process repeated 3 to 5 times. Then, 100 μL of enzyme-labeled antibody was added to each well, followed by incubation at 18 to 26°C for 20 min, and the washing process was repeated. Next, 100 μL of 3,3′,5,5′-tetramethylbenzidine (TMB) substrate was added to each well and incubated at 18 to 26°C for 15 min. Finally, 50 μL of stop solution was added to each well to terminate the color reaction. The optical density (OD) values of the tested sample and the control were measured and recorded at an absorbance of 650 nm. The *S*/*N* value was calculated by dividing the OD value of the sample (*S*) by the OD value of the negative control (*N*). If the *S*/*N* value was >0.7, the sample was considered negative; if 0.6 < *S*/*N* ≤ 0.70, the sample was deemed suspicious; and if the *S*/*N* value was ≤0.60, the sample was considered positive. If the test result of the sample remained suspicious after repeated detection, the sample was discarded.

### Data analysis.

All collected data were inputted and organized in Excel (Excel 2007, Microsoft, USA). Utilizing the Clopper-Pearson method (29), the EpiR package (version 2.0.43) was employed to calculate the positivity rate and 95% CI of PRV gE seroprevalence in pig herds by using R software (R Core Team 2020) (30, 31). Concurrently, the Pearson chi-square test was applied to analyze the differences in PRV gE seroprevalence among various provinces and pig categories (32). A pig farm with at least one gE antibody-positive sample was considered PRV positive. A pig farm's PRV serological status was treated as a dichotomous variable (PRV-positive or -negative farm). Single variable effects on the PRV serological status in pig farms were examined using a univariate logistic regression model. Variables with a *P* value of <0.1 in the univariate logistic regression analysis were selected for subsequent multivariate logistic regression models using a backward stepwise regression method (33). The variance inflation factor was utilized to detect multicollinearity among variables (34). If multicollinearity was present, variables with biological significance were retained in the final model. These analyses were performed using the R project's “car” package (version 3.1-1) (35).

SaTScan version 9.6 software was utilized to predict the spatial-temporal aggregation distribution of high PRV gE seroprevalence based on the Bernoulli model (36, 37). The numbers of PRV gE antibody-positive and -negative samples in each pig farm were considered the experimental and control groups, respectively. Time aggregation was conducted at the month level, encompassing the entire experiment period from December 2017 to May 2021.

The time series model of PRV gE seroprevalence was established using the autoregressive moving average (ARMA) method. Due to the novel coronavirus outbreak (COVID-19), pig blood samples could not be collected in February and March 2020, resulting in missing data. Therefore, R package “mice” (version 3.14.0) was employed to perform multiple imputations on the missing data, generating a complete data set for subsequent model building (38). The model with the smallest AIC value was chosen as the final model (39).

Epidemic trend analysis of PRV gE seroprevalence was conducted using the following steps. First, based on the established PRV time series model, @RISK software (version 7.0) was used to simulate the distribution histogram of PRV gE seroprevalence each month (June 2021 to May 2023). Then, the PRV gE seroprevalence of adjacent months was subtracted to obtain the monthly average change rate, averaged across all monthly change rates. Finally, the PRV gE seroprevalence distribution histogram's monthly average change rate was obtained. A total of 10,000 Monte Carlo simulations were performed to estimate the monthly average change rates of PRV gE seroprevalence using the Latin hypercube sampling method (40–42). Additionally, maps were drawn using ArcGIS 10.7 (ESRI, USA) software.

### Data availability.

All raw data supporting the findings of this study are available by contacting the corresponding author. Data are not publicly available due to privacy and ethical restrictions.
